# Supplementary material for: Control of quantum electrodynamical processes by shaping electron wavepackets
Source: Nat Commun. 2021 Mar 17;12:1700. doi: 10.1038/s41467-021-21367-1 (PMC7969958; doi:10.1038/s41467-021-21367-1)
Supplement: Supplementary file 1 — Supplementary Information [file 41467_2021_21367_MOESM1_ESM.pdf]

Supplementary Information: Control of quantum electrodynamical processes by shaping electron wavepackets

Liang Jie Wong[1,*], Nicholas Rivera[2], Chitraang Murdia[2], Thomas Christensen[2],

John D. Joannopoulos[2], Marin Soljačić[2] and Ido Kaminer[3,*]

[1]School of Electrical and Electronic Engineering, Nanyang Technological University, Singapore 639798, Singapore.
[2]Department of Physics, Massachusetts Institute of Technology, Cambridge, MA 02139, USA.
[3]Department of Electrical Engineering, Technion, Haifa 32000, Israel.

[*]Email: liangjie.wong@ntu.edu.sg, kaminer@technion.ac.il

S1. Interference in quantum electrodynamic processes

The purpose of this section is to elucidate the physics of interference arising from electron waveshaping, and how it leads to the enhancement of radiative processes such as atomic Bremmstrahlung (Fig. 2 of the main text) and undulator bremsstrahlung (Fig. 3 of the main text). The ability to control quantum electrodynamical (QED) processes through electron waveshaping arises directly from the wave-like nature of the input and output particles in QED processes. This wave-like nature of input and output allows the quantum mechanical transition amplitudes of the processes themselves to constructively or destructively interfere, where each process is associated with exactly one input momentum state. As the emission rate is the square of the total amplitude, this enables interference to play a decisive role in the overall emission rate. As such, the shaping of an input electron wavepacket, which consists of multiple momentum states, can be used to control and engineer interferences in the processes for which the electron wavepacket is an input. A more quantitative description of this phenomenon for the case of two input momentum states $|i_1\rangle$ and $|i_2\rangle$ is given via Eqs. (1-3) of the main text. There, we see that the right choice of shaped electron input can change the cross section from an incoherent cross section $\propto |M_1|^2 + |M_2|^2$ (Eq. (2)) to a coherent cross section $\propto |M_1 + M_2|^2$ (Eq.(3)), where $M_{1,2}$ are the scattering amplitudes for the respective

input momentum states modulo the delta distributions. The difference between the coherent and incoherent cross sections is the cross term $2\mathrm{Re}\{M_1^* M_2\}$, which is generally nonzero and can be substantial compared to the incoherent cross section.

Figs. S1 and S2 illustrate this phenomenon using the atomic bremsstrahlung example in Fig. 2g-j, and the undulator bremsstrahlung example in Fig. 3c-f. By breaking down the overall cross section $|A+B|^2$ into the constituent terms $|A|^2$, $|B|^2$ and cross term $2\mathrm{Re}\{A^* B\}$, we see the important role of the cross term in bringing about the enhancement in the final output.

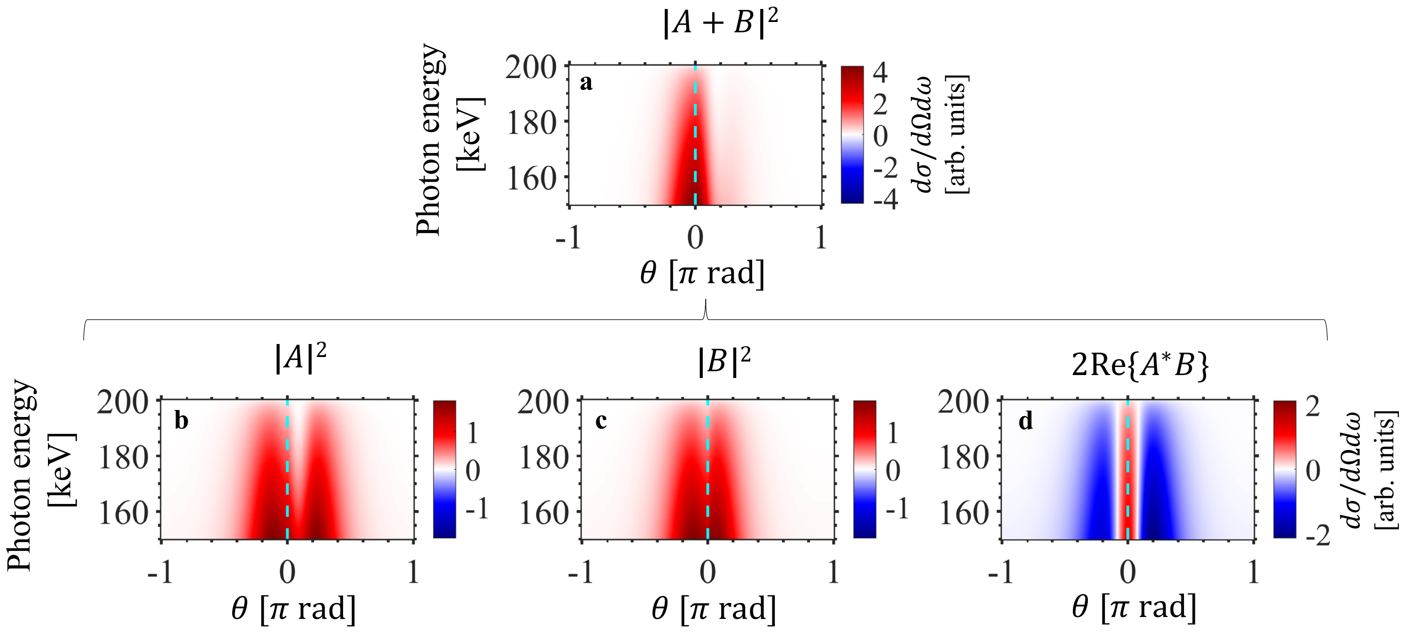

Figure S1. Illustration of quantum interference arising from a two-state electron input, resulting in enhanced atomic bremsstrahlung. The parameters of this study are exactly those used in Fig. 2g-j of the main text. (a) shows Fig. 2i rendered in a different colormap, where quantum interference between the constituent processes results in an output cross section $|A+B|^2$ (A and B correspond to the transition amplitudes from the two input states respectively). To understand the contrast between the coherent output $|A+B|^2$ vs. the incoherent output $|A|^2+|B|^2$, we break $|A+B|^2 = |A|^2 + |B|^2 + 2\mathrm{Re}\{A^* B\}$ down into its constituent terms in (b-d). We see that the cross term shown in (d) is instrumental in the suppression of off-axis radiation, as well as in the enhancement of on-axis radiation, in the final result (a). This represents destructive interference off-axis, but constructive interference on-axis between the processes A and B.

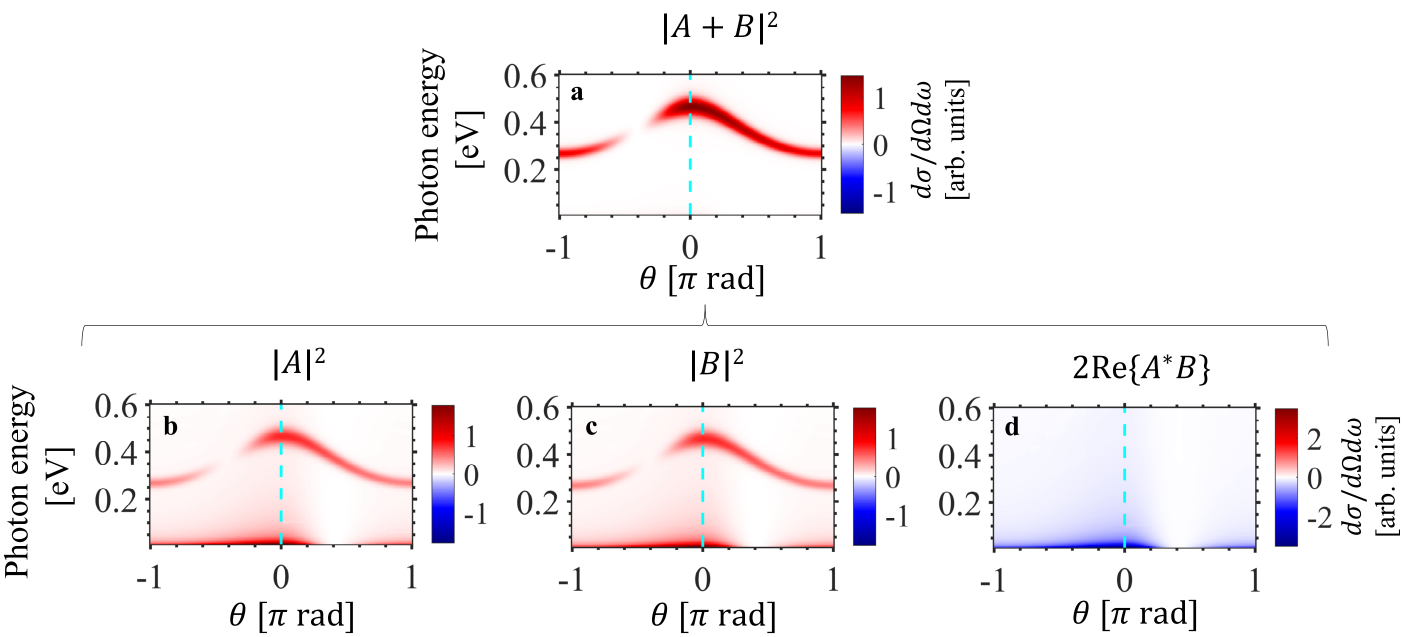

Figure S2. Illustration of quantum interference arising from a two-state electron input, resulting in enhanced undulator bremsstrahlung. The parameters of this study are exactly those used in Fig. 3c-f. (a) shows Fig. 3e rendered in a different colormap, where quantum interference between the constituent processes results in an output cross section $|A+B|^2$ (A and B correspond to the transition amplitudes from the two input states respectively). To understand the contrast between the coherent output $|A+B|^2$ vs. the incoherent output $|A|^2+|B|^2$, we break $|A+B|^2=|A|^2+|B|^2+2\mathrm{Re}\{A^*B\}$ down into its constituent terms in (b-d). We see that the cross term shown in (d) is instrumental in the suppression of low-frequency radiation in the final result (a). This represents destructive interference at lower frequencies, but not at higher frequencies near the desired peak, between the processes A and B.

S2. Proposed scheme for experimental realization of quantum interference in bremsstrahlung

The purpose of this section is to present calculations towards the design of experimental demonstrations for control of radiative processes via electron waveshaping. Both examples of atomic bremsstrahlung and undulator bremsstrahlung, presented in Figs. 2 and 3 respectively in the main text, can be experimentally verified based on the schematic shown in Fig. S3. In Fig. S3, an electron plane wave impinges on a biprism, which consists of two parallel grounded plates with a fine filament between them. The filament has a positive potential, resulting in an electrostatic field that splits the

incident plane wave into two deflected plane waves. The use of the electrostatic biprism for this very purpose was demonstrated in Refs. [1, 2], where the interference fringes of the resulting electron wave superposition were measured. The generation of crossed electron beams using biprisms is also well-known in electron holography [3], where carrier fringe spacings on the order of several picometers have been achieved [4].

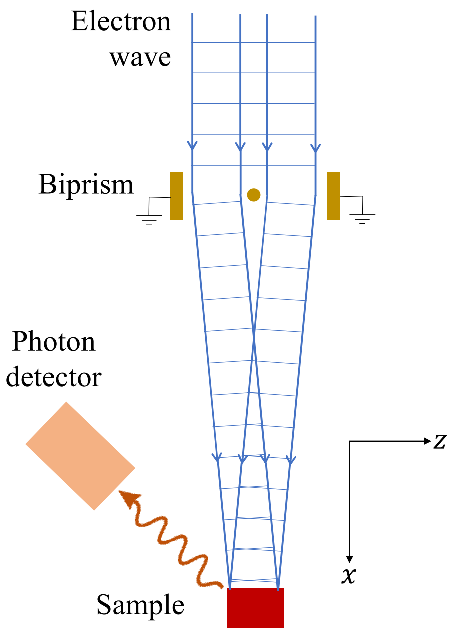

Figure S3. Experimental schematic for demonstrating control of bremsstrahlung processes via quantum interference from shaped electron wavepackets. An electron plane wave impinges on a biprism, which consists of two parallel grounded plates with a fine filament between them. The biprism converts the plane wave into two deflected plane waves, which is the input assumed in the multi-state electron wave examples in Figs. 2 and 3 of the main text. The sample is placed within the region of overlap between the two deflected plane waves, and photons emitted (brown wiggly line) from the bremsstrahlung process are collected by a photon detector.

Instead of an electrostatic biprism, one may shape the electron wavepacket using single crystal thin films [5], binary amplitude masks [6,7], patterned thin SiN membranes [8], electron-photon interactions [9], electrostatic elements [10,11], or magnetic vortices [12] and needles [2]. Many of

these schemes go beyond the simple deflection of an electron wave, and testify to the wide range of electron wavepackets and waveshaping methods available.

One may theoretically estimate the deflection of the electron beam by the biprism when the fields of the positive filament are well-approximated by that of an infinitely long charged wire, and the deflection of the electron beam in the transverse direction x (perpendicular to its direction of original travel, which we denote z) is nonrelativistic. As a result, the electron beam originally traveling at a momentum $\hbar k_z$ in the z-direction, where $\hbar$ is the reduced Planck's constant, is deflected by a momentum $\hbar k_x$ in the x-direction, whose magnitude is given by [1]

$$|k_x| = \left| \frac{me}{\hbar^2 k_z} \int_{-\infty}^{\infty} \left(\frac{\partial V(x, z')}{\partial x} \right)_{x=a} dz' \right| = \frac{\pi e V_a}{\hbar v_z \ln\left(\frac{b}{a}\right)}, \tag{S1}$$

where m is electron mass, e the elementary charge, a the radius of the filament, $b > a$ the separation between grounded plate and filament, $V_a > 0$ the potential of the filament, and $v_z > 0$ the speed of the electron in z. The second equality is achieved using a potential V given by

$$V(x, z) = V_a \left[\frac{\ln\left(\frac{\sqrt{x^2 + z^2}}{b} \right)}{\ln\left(\frac{a}{b}\right)} \right], \tag{S2}$$

which is consistent with the potential being V_a at the surface of the filament ($x = \pm a, z = 0$), and the potential being 0 at the grounded plates ($x = \pm b, z = 0$). For a 20 keV electron beam, Eq. (S1) shows that a deflection angle $\theta_d = \arctan\,(k_x/k_z)$ of 8.7×10^{-5} rad is achieved with $V_a = 10\,V$, $a = 0.5\,\mu$m, $b = 5$ mm. Under the small angle approximation, Eq. (S1) shows that θ_d scales linearly with V_a, allowing larger deflection angles to be achieved with higher filament potentials. Still larger deflections may be obtained through the use of an appropriate focusing element placed after the

biprism. The focusing element will compress the interference pattern fringes, effectively increasing the value of k_x and hence the deflection angle θ_d.

Fig. S4 shows the differential cross sections for a 5 keV electron wavepacket incident on a Tungsten (W) atom, for a spin-polarized (Fig. S4a-e) [13-16] and unpolarized (Fig. S4f-j) electron source. In the spin-polarized case, we notice a distinct change in emission pattern when going from the incoherent case in Fig. S4b, to the coherent case in Fig. S4c. Furthermore, the cross section drops dramatically, indicating the destructive interference that occurs when the electron waves of opposite phase generate atomic bremsstrahlung at such low incident angles. When the electron waves are of the same phase (Fig. S4d), we see a constructive interference effect taking place, that enhances the radiation cross section to levels beyond the unshaped and shaped incoherent cases in Figs. S4a and b respectively. The relative phases of the electron waves can be controlled, for instance, through more sophisticated designs that use the Aharanov-Bohm effect [2,17,18]. However, regardless of the relative phases between the two input electron states, we predict a distinctive and measurable change in the emitted photon rate and emission profile, which occurs when going from the unshaped (or shaped incoherent) to the shaped coherent cases. In the unpolarized case (Figs. S4f-j), the *shapes* of the emission profile are similar in all cases, but the measured photon emission rate are substantially different. Due to destructive (constructive) interference, the shaped coherent case Fig. S4h (Fig. S4i) has a markedly different photon emission rate compared with the unshaped as well as shaped incoherent cases. These comparisons provide strong evidence for the ability to control radiative processes through quantum interference arising from electron waveshaping.

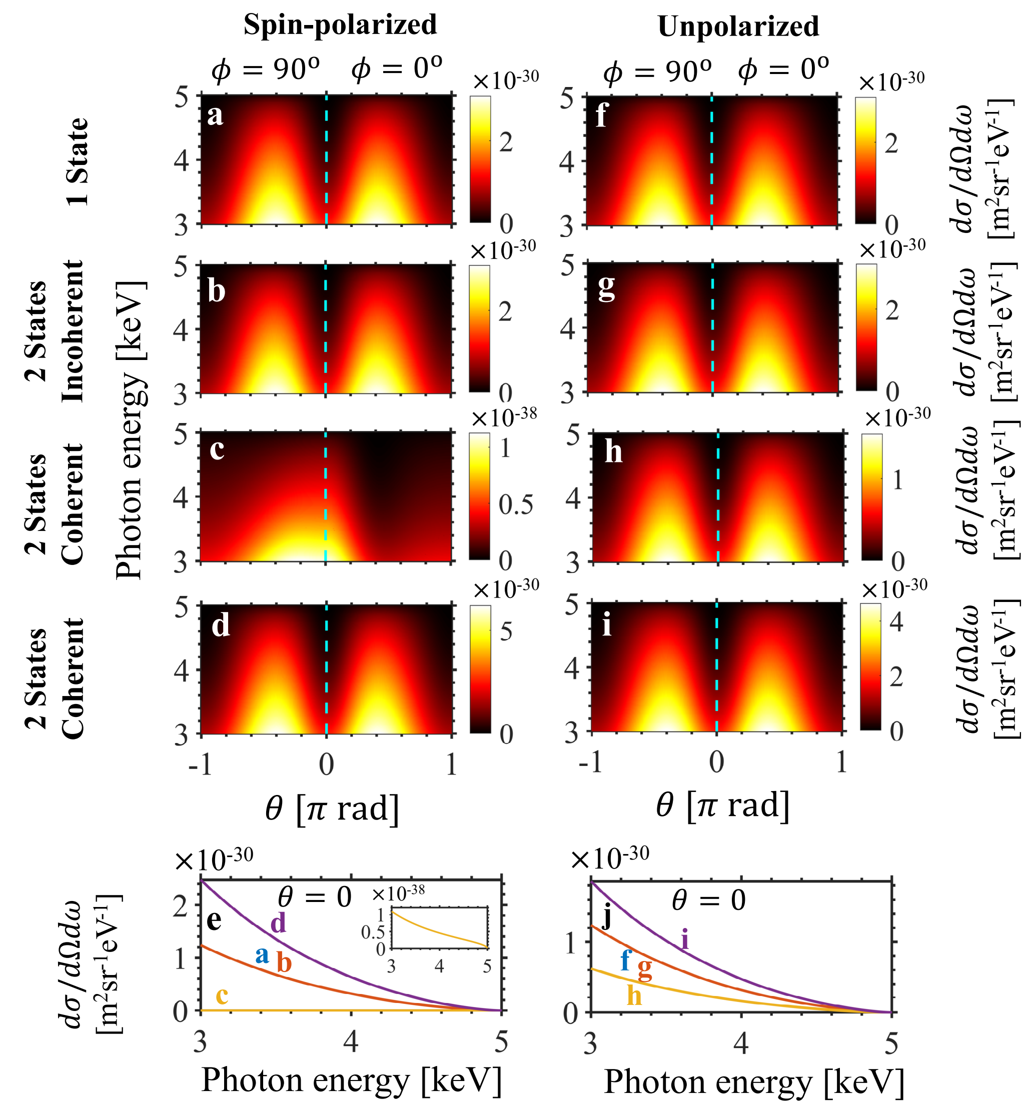

Figure S4. Atomic bremsstrahlung in a Tungsten (W) atom subject to a 5 keV electron wavepacket at low deflection angles. In the typical atomic bremsstrahlung scenario, a single momentum state electron scatters off a W atom and emits radiation. (a) shows the output photon differential cross section from this process for a 5 keV electron, with a single z-directed input electron state. (b)-(d) show the differential cross sections for the case of two input electron states oriented at $\theta_d = 26.2\ \mu$rad with respect to the z-axis. Quantum interference between the constituent processes in (c) causes a near-cancellation of the output profile, as well as a change in its shape to become more directional and peaked on-axis. In (c), the electron states are of opposite phase. Making the electron states of the same phase in (d) leads instead to an enhanced emission. The on-axis profile of (a-d) is shown in (e). In (a-e), the input electron states are spin-polarized in the same direction (upwards). The results in (f-j) correspond to the same scenarios in (a-e) respectively, except (f-j) considers an unpolarized electron source by averaging over all input spin combinations. A significant change in the cross section, which can be experimentally measured, still occurs when going from the unshaped to shaped coherent scenarios. In (e,j) the blue and red curves overlap exactly. Unless otherwise stated, all parameters are the same as Fig. 2 of the main text.

The total cross sections in the photon energy range shown in Figs. S4a-d are 4.29×10^{-19} m^2, 4.29×10^{-19} m^2, 8.77×10^{-28} m^2 and 8.58×10^{-19} m^2 respectively, for electron interaction with a single atom. Using the Kanaya-Okayama formula [19] to estimate the penetration depth of a 5 keV electron beam into Tungsten (about 80 nm), we estimate that the average electron fired normally into the sample would encounter about 300 atoms. We consider an electron beam radius of 0.5 μm and an average current of 1 nA, and calculate for Figs. S4a-d the following photon emission rates (note the plunge in the bremsstrahlung output in panel c): 1×10^6 photons/s, 1×10^6 photons/s, 2×10^{-3} photons/s, and 2×10^6 photons/s, respectively. The total cross sections in Figs. S4f-i are 4.29×10^{-19} m^2, 4.29×10^{-19} m^2, 2.14×10^{-19} m^2 and 6.43×10^{-19} m^2 respectively, for a single atom. For a 5 keV electron beam of radius 0.5 μm and average current 1 nA, this corresponds to photon emission rates of 1×10^6 photons/s, 1×10^6 photons/s, 5×10^5 photons/s and 1.5×10^6 photons/s respectively. These X-ray photons can be measured via energy dispersive X-ray spectroscopy (EDX) or wavelength dispersive X-ray spectroscopy (WDX).

References

[1] Tonomura, A., Endo, J., Matsuda, T. Kawasaki, T. & Ezawa, H. Demonstration of single-electron buildup of an interference pattern. Am. J. Phys 57, 117-120 (1989).

[2] Guzzinati, G., Béché, A., Lourenço-Martins, H., Martin, J., Kociak, M. & Verbeeck, J. Probing the symmetry of the potential of localized surface plasmon resonances with phase-shaped electron beams. Nat. Commun. 8, 14999 (2017).

[3] McCartney, M. R., Dunin-Borkowskib, R. E., & Smith, D. J. Quantitative measurement of nanoscale electrostatic potentials and charges using off-axis electron holography: Developments and opportunities. Ultramicroscopy 203, 105–118 (2019).

[4] Harada, K. & Tonomura, A. High-resolution observation by double-biprism electron holography J. Appl. Phys. 96, 6097 (2004).

[5] Ru, Q., Osakabe, N., Endo, J., & Tonomura, A. Electron holography available in a non-biprism transmission electron microscope. Ultramicroscopy 53, 1-7 (1994).

[6] McMorran, B. J., Agrawal, A., Anderson, I. M., Herzing, A. A., Lezec, H. J., McClelland, J. J., & Unguris, J. Electron vortex beams with high quanta of orbital angular momentum. Science 331, 192-195 (2011).

[7] Verbeeck, J., Tian, H. & Schattschneider, P. Production and application of electron vortex beams. Nature 467, 301-304 (2010).

[8] Shiloh, R., Lereah, Y., Lilach, Y., & Arie, A. Sculpturing the electron wave function using nanoscale phase masks. Ultramicroscopy 144, 2631 (2014).

[9] Reinhardt, O., & Kaminer, I. Theory of Shaping Electron Wavepackets with Light, ACS Photonics, Just Accepted Manuscript

[10] Duchamp, M., Girard, O., Pozzi, G., Soltner, H., Winkler, F., Speen, R., Dunin-Borkowski, R.-E., & Cooper, D. Fine electron biprism on a Si-on-insulator chip for off-axis electron holography. Ultramicroscopy 185, 81–89 (2018).

[11] Pozzi, G. Lu, P.-H., Tavabi, A. H., Duchamp, M., & Dunin-Borkowski, R. E. Generation of electron vortex beams using line charges via the electrostatic Aharonov-Bohm effect Ultramicroscopy 181, 191–196 (2017).

[12] Zheng, C., Petersen, T. C., Kirmse, H., Neumann, W., Morgan, M. J., & Etheridge. J. Axicon Lens for Electrons Using a Magnetic Vortex:The Efficient Generation of a Bessel Beam. Phys. Rev. Lett. 119, 174801 (2017).

[13] Pierce, D. T., Celotta, R. J., Wang, G.-C., Unertl, W. N., Galejs, A., Kuyatt, C. E., & Mielczarek, S. R. The GaAs spin polarized electron source. Rev. Sci. Instrum. 51, 478 (1980).

[14] Mamaev, Y. A., Gerchikov, L. G., Yashin, Y. P., Vasilyev, D. A., Kuzmichev, V. V., Ustinov, V. M., Zhukov, A. E., Mikhron, V. S., & Vasiliev, A. P. Optimized photocathode for spin-polarized electron sources. Appl. Phys. Lett. 93, 081114 (2008).

[15] Lampel, G., & Weisbuch, C. Proposal for an efficient source of polarized photoelectrons from semiconductors. Solid State Commun. 16, 877 (1975).

[16] Pierce, D. T., Meier, F., & Zürcher, P. Negative electron affinity GaAs: A new source of spin-polarized electrons. Appl. Phys. Lett. 26, 670 (1975).

[17] Tonomura, A., Osakabe, N., Matsuda, T., Kawasaki, T., Endo, J., Yano, S., & Yamada, H. Evidence for Aharonov-Bohm effect with magnetic field completely shielded from electron wave. Phys. Rev. Lett. 56, 792 (1986).

[18] Pozzi, G. Lu, P.-H., Tavabi, A. H., Duchamp, M., & Dunin-Borkowski, R. E. Generation of electron vortex beams using line charges via the electrostatic Aharonov-Bohm effect Ultramicroscopy 181, 191–196 (2017).

[19] Kanaya, K., & Okayama, S. Penetration and energy-loss theory of electrons in solid targets. J. Phys. D 5, 43 (1972).
