# Supplementary material for: Control of quantum electrodynamical processes by shaping electron wavepackets
Source: Nat Commun. 2021 Mar 17;12:1700. doi: 10.1038/s41467-021-21367-1 (PMC7969958; doi:10.1038/s41467-021-21367-1)
Supplement: Supplementary file 3 — Source Data [file 41467_2021_21367_MOESM3_ESM.zip › Fig3/data_info_Fig3.docx]

The data in this folder correspond to the panels presented in Fig. 3.

All data is in the respective units and normalizations as presented in the figure.

The data files map to the various components of the figure as follows:

x_3cde: x-axis of Figs. 3c-e

y_3cde: y-axis of Figs. 3c-e

Fig3c: Data for Fig. 3c

Fig3d: Data for Fig. 3d

Fig3e: Data for Fig. 3e

x_3ghi: x-axis of Figs. 3g-i

y_3ghi: y-axis of Figs. 3g-i

Fig3g: Data for Fig. 3g

Fig3h: Data for Fig. 3h

Fig3i: Data for Fig. 3i
