# Supplementary material for: Control of quantum electrodynamical processes by shaping electron wavepackets
Source: Nat Commun. 2021 Mar 17;12:1700. doi: 10.1038/s41467-021-21367-1 (PMC7969958; doi:10.1038/s41467-021-21367-1)
Supplement: Supplementary file 3 — Source Data [file 41467_2021_21367_MOESM3_ESM.zip › FigM2/data_info_FigM2.docx]

The data in this folder correspond to the panels presented in Fig. M2.

All data is in the respective units and normalizations as presented in the figure.

The data files map to the various components of the figure as follows:

x_M2l: x-axis for leftmost panel

y_M2l: y-axis for leftmost panel

M2l: data for leftmost panel

x_M2mr: x-axis for middle and rightmost panels

y_M2mr: y-axis for middle and rightmost panels

M2m: data for middle panel

M2r: data for rightmost panel
